# Supplementary material for: Development and Evaluation of a Set of Spike and Receptor Binding Domain-Based Enzyme-Linked Immunosorbent Assays for SARS-CoV-2 Serological Testing
Source: Diagnostics (Basel). 2021 Aug 20;11(8):1506. doi: 10.3390/diagnostics11081506 (PMC8393265; doi:10.3390/diagnostics11081506)
Supplement: Supplementary file 1 [file diagnostics-11-01506-s001.zip › diagnostics-1288403-supplementary.pdf]

diagnostics

Supplementary Materials

Supplementary Table S1. Repeatability of the minimum, maximum, and average values of the S1 and RBD IgG assays for the positive and negative optimization sets. The statistics for the minimum and maximum values are the result of six measurements.

S1				RBD			
Positive Samples (*n* = 15)				Positive Samples (*n* = 15)			
**Value**	**Average**	**Standard Deviation**	**CV (%)**	**Value**	**Average**	**Standard Deviation**	**CV (%)**
Min	1.42	0.03	1.51	Min	0.6	0.02	1.34
Max	2.19	0.14	6.81	Max	1.96	0.08	7.63
Average	1.91	0.19	10.21	Average	1.33	0.38	28.28
Negative Samples (*n* = 15)				Negative Samples (*n* = 15)			
**Value**	**Average**	**Standard Deviation**	**CV (%)**	**Value**	**Average**	**Standard Deviation**	**CV (%)**
Min	0.08	0	3.28	Min	0.14	0.01	2.77
Max	0.36	0.02	10.81	Max	0.38	0.04	12.08
Average	0.14	0.07	51.53	Average	0.29	0.07	23.66

Supplementary Table S2. Serum samples description. The fifth column lists the time (in days) elapsed between the onset of the symptoms and the RT-PCR test. The sixth column lists the time (in days) elapsed between the RT-PCR test and the collection of the serum sample. The last column is the sum of days in the fifth and sixth columns. Notice that in group 3, another serum sample was taken the day of the RT-PCR test (fifth column).

Sample ID	Group	Age	Sex	Days from Onset (PCR test)	Days from PCR (Serum Sample)	Days from Onset (Serum Sample)
1	1	27	M	5	10	15
2	1	42	M	8	13	21
3	1	27	M	2	13	15
4	1	35	M	4	14	18
5	1	27	M	4	14	18
6	1	27	F	5	15	20
7	1	30	M	5	15	20
8	1	28	M	2	17	19
9	1	27	M	6	17	23
10	1	37	M	4	20	24
11	1	26	M	1	21	22
12	1	55	M	4	22	26
13	1	39	M	2	23	25
14	1	23	M	3	24	27
15	1	51	M	8	24	32
16	1	39	F	8	27	35
17	1	49	F	6	27	27
18	1	37	F	5	27	32
19	1	39	M	5	30	35
20	1	48	F	4	31	35
21	1	35	F	0	42	42
22	2	40	F	4	18	22
23	2	29	F	14	26	40
24	2	33	M	0	27	27
25	2	34	M	11	29	40
26	2	37	M	8	30	38

27	2	23	M	20	31	51
28	2	22	F	6	31	37
29	2	26	M	0	31	31
30	2	26	F	4	31	35
31	2	43	F	6	32	38
32	2	25	M	18	34	52
33	2	29	M	9	34	43
34	2	41	M	0	35	35
35	2	44	F	5	35	40
36	2	46	M	12	35	47
37	2	38	F	3	38	41
38	2	55	M	2	62	64
39	3	26	M	8	32	40
40	3	50	M	12	32	44
41	3	40	M	10	29	39
42	3	23	M	4	32	36
43	3	47	M	7	25	32
44	3	26	F	4	31	35
45	3	61	F	2	30	32
46	3	65	M	8	30	38
47	3	37	M	11	28	39
48	3	36	M	5	29	34
49	3	58	F	11	28	39
50	3	48	M	1	44	45

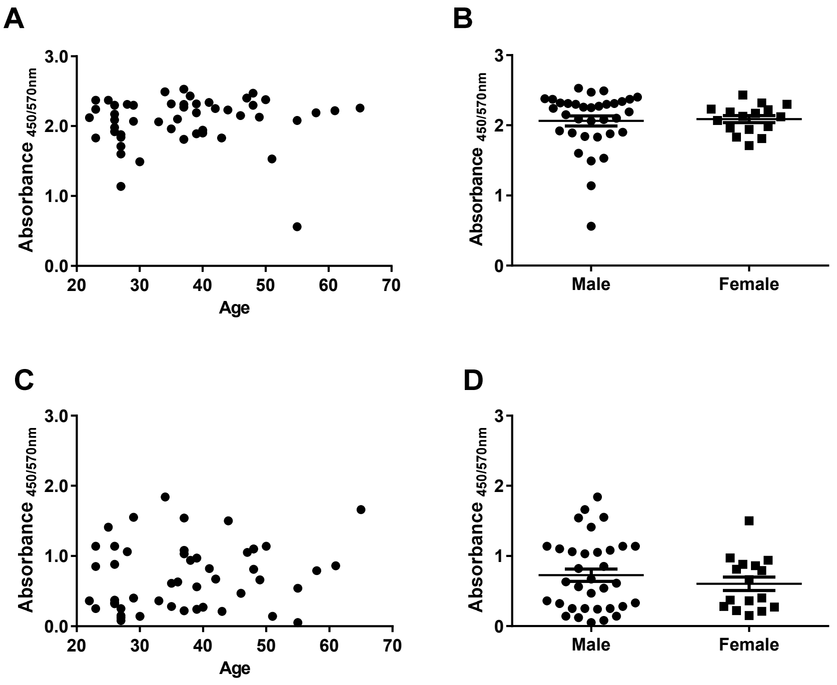

Supplementary Figure S1. Detection of anti-RBD IgG serum sample by age and sex. Samples were analyzed at a dilution of 1:100 (**A**,**B**) and 1:3000 (**C**,**D**). There was no correlation between age (left panels) and IgG anti-RBD in serum in any dilution, according to a Pearson correlation analysis ($p < 0.05$). A Student *t*-test for B and D ($p < 0.05$) indicated a non-significant difference between IgG anti-RBD and sex.
